# Diagnostic Criteria, Differential Diagnosis, and Treatment of Minor Motor Activity and Less Well-Known Movement Disorders of Sleep

**DOI:** 10.1007/s11940-019-0543-8

**Published:** 2019-01-19

**Authors:** Ambra Stefani, Birgit Högl

**Affiliations:** Department of Neurology, Medical University of Innsbruck, Anichstrasse 35, 6020 Innsbruck, Austria

**Keywords:** EFM, Neck myoclonus, Isolated RBD, Idiopathic RBD, Prodromal RBD

## Abstract

**Purpose of review:**

Sleep-related movement disorders (SRMD) include several different motor activities during sleep. Few of them are well known and well classified, whereas others are minor motor disorders of sleep which are neither thoroughly characterized and classified nor have been extensively investigated to clarify their pathogenesis and clinical relevance. This review will focus on those minor sleep-related movement disorders.

**Recent findings:**

Before diagnosing periodic limb movement (PLM) disorder in patients with PLM during polysomnography, other disorders associated with PLM need to be excluded, namely restless legs syndrome (RLS), narcolepsy, REM sleep behavior disorder (RBD), and sleep-related breathing disorder. For the diagnosis of propriospinal myoclonus at sleep-onset, multi-channel surface electromyography recording during polysomnography is required and a possible psychogenic origin of the movement disorder has to be considered. Excessive fragmentary myoclonus (EFM) does not require symptomatic treatment, but further evaluation is suggested as electrophysiological abnormalities are present in 50% of cases. Nine percent of healthy sleepers meet the criteria for EFM, raising the question if current, arbitrarily defined, cutoffs are valid. Hypnagogic foot tremor, rhythmic feet movements, alternating leg muscle activation, and high-frequency leg movements are somewhat overlapping minor motor activities during sleep which may exist on their own or represent stereotyped movements to relieve RLS-like symptoms. Neck myoclonus is probably a physiological phenomenon related to REM twitching. RBD is formally a parasomnia but a relevant differential diagnosis when evaluating sleep-related movement disorders. In particular, prodromal RBD is characterized by electromyographic and behavioral findings on video-polysomnography which needs to be differentiated by minor sleep-related movement disorders.

**Summary:**

Minor SRMD beyond the well-known main motor disorders of sleep should be correctly diagnosed, distinguished from differential diagnosis, and understood in their potential clinical relevance, in order also to start an appropriate treatment if needed.

## Introduction

Most physicians associate sleep-related movement disorders (SRMD) with restless legs syndrome (RLS), periodic leg movements during sleep (PLMS), and parasomnias with abnormal movements and behaviors during sleep, such as REM sleep behavior disorder (RBD). According to the ICSD-3 [1], SRMD include RLS, periodic limb movement disorder (PLMD), sleep-related leg cramps, sleep-related bruxism, sleep-related rhythmic movement disorder, benign sleep myoclonus of infancy, and newly in the current edition propriospinal myoclonus at sleep onset. Moreover, within the ICSD-3 category of SRMD, the subcategory of “isolated symptoms and normal variants” includes excessive fragmentary myoclonus (EFM), hypnagogic foot tremor (HFT), and alternating leg muscle activation (ALMA), and sleep starts [1] (Table 1). Rhythmic feet movements (RFM) and high-frequency leg movements (HFLM) are not included in this category. Another frequent SRMD, neck myoclonus during (REM) sleep or head jerks, is also yet not classified in the ICSD-3. RBD is listed among the REM sleep parasomnias, but as its main feature are increased EMG activity, jerks, and movements during REM sleep, it represents an important differential diagnosis for some SRMD.

**Table 1:** ICSD-3 definite sleep-related movement disorders [1]

ICSD-3 definite sleep-related movement disorders	
• Restless legs syndrome (RLS)	
• Periodic limb movement disorder	
• Propriospinal myoclonus at sleep onset	
• Sleep related leg cramps	
• Sleep related bruxism	
• Sleep related rhythmic movement disorder	
• Benign sleep myoclonus of infancy	
• Propriospinal myoclonus at sleep onset	
• Sleep related movement disorder due to a medical disorder	
• Sleep related movement disorder due to a medication or substance	
• Sleep related movement disorder, unspecified	
• Isolated symptoms and normal variants: - Excessive fragmentary myoclonus - Hypnagogic foot tremor and alternating leg muscle activation - Sleep starts	

While the most well-known sleep disorders with abnormal motor activity during the night, such as RLS and RBD, are the focus of multiple reviews [2•, 3•, 4•, 5•, 6, 7, 8•, 9, 10], the other motor disorders of sleep are often considered less frequent, less important, or not clinically relevant. This leads to the fact that some of them are frequently missed or misinterpreted in their meaning and implications or confounded in differential diagnosis.

The aim of this review is to address those minor sleep-related movement disorders which are often misdiagnosed or misinterpreted, focusing on their diagnostic criteria, differential diagnosis, prevalence (as far as data are available), clinical implications, meaning, and, as far as it is known, treatment.

## Periodic limb movement disorder

### Overview

Periodic limb movement disorder (PLMD) is a frequently given diagnosis based on PSG only, but in many cases it could be misattributed, specifically in patients where PLMS are seen in PSG and no further evaluations regarding other sleep pathologies which frequently go along with PLM is undertaken.

### Diagnostic criteria

According to the ICSD-3[1], diagnosis of PLMD requires documentation in the PSG of PLMS with an index of > 5/h in children and > 15/h in adults. Additionally, PLMS must cause clinically significant sleep disturbance or impairment in various relevant areas of functioning and cannot be better explained by another current sleep, medical, or neurological disorder such as RLS, narcolepsy, or RBD. Accordingly, if PLMS are seen in PSG, all these abovementioned other conditions need to be actively excluded before making a diagnosis of PLMD.

### Differential diagnosis

The first step in a patient with PLMS should be an accurate clinical interview aiming to RLS symptoms, as RLS is a frequent condition and PLMS are present in more than 80% of patients with RLS [1, 11]. Interestingly, the presence of PLMS even in the absence of RLS has been associated with RLS risk genes [12, 13••], suggesting common underlying pathogenetic mechanisms in both conditions. If PLMS are found in PSG in a patient complaining about excessive daytime sleepiness (EDS), EDS may not be attributed to the PLMD, but narcolepsy has to be excluded, as PLMS are frequent (up to 75%) in patients with narcolepsy [1, 14]. In patients with PLMS and dream enactment, the clinical interview should also focus on clinical history of RBD. PLMS are common in RBD (about 70%), particularly during REM sleep, which is unusual in other conditions. Moreover, an accurate video-PSG review evaluating visible movements and the presence of REM sleep without atonia (RWA) is needed in these cases, in order not to misdiagnose patients with RBD as this condition has important implications representing an early stage alpha-synucleinopathy [5•]. Additionally, PLMS are an important differential diagnosis in case of suspected RBD, as arousals with abnormal behaviors may follow PLMS, thus mimicking RBD symptoms [15]. PLMS may also be associated with sleep-related breathing disorder (SRBD) [16–18]. In case of coexistence of PLMS and SRBD, the latter should be treated first and the presence of PLMS should be reassessed after adequate treatment of the SRBD.

### Prevalence

In 100 healthy sleepers who underwent video-PSG, in which the presence of any sleep disorder including RLS was excluded by an expert interview, mean PLMS index was 9.2/h, with a range of 0–62 [19]. In another general population study, a PLMS index > 15/h was reported in 28.6% of subjects. However, 25.3% of these subjects reported RLS symptoms as evaluated by questionnaires [20]. Still, PLMS can be present during sleep as a simple polysomnographic finding without RLS and without indicating PLMD.

As for a correct diagnosis of PLMD many other conditions need to be excluded, and diagnosis requires that PLMS cause clinically significant sleep disturbance or impairment in various relevant areas of functioning, its real prevalence is not known and it has even been questioned if PLMD really exists.

### Clinical implications/meaning

True PLMD (as opposed to PLMS alone) is characterized by definition by significant sleep disturbances, mainly sleep onset or maintenance problems. However, EDS is usually absent in patients with PLMD [1]. Apart from causing sleep disturbances in the context of PLMD, PLMS have been associated with the risk of cardiovascular events [21–25]. One possible explanation for this association could be the PLMS-related activation of the sympathetic nervous system [22]. However, the relationship between PLMS and cardiovascular events and the potential underlying pathogenetic mechanisms still need to be systematically evaluated [26••].

### Treatment

Studies evaluating treatment of PLMD are scarce and medications that are useful for idiopathic RLS have not been sufficiently investigated in the setting of PLMD. Dopaminergic treatment is not indicated in PLM alone and it has been reported that dopaminergic treatment of PLM alone may over time induce RLS [27]. Few studies in PLMD reported clonazepam and valproate to be effective on sleep quality but not on PLMS index, whereas melatonin reduced PLMS index [28]. However, randomized controlled trials on PLMD are lacking.

## Propriospinal myoclonus at sleep onset

### Overview

Propriospinal myoclonus (PSM) with mainly axial jerks at the sleep-wake transition was described in 2001 by the Bologna group [29]. They observed and described myoclonic activity arising during the relaxed wakefulness (predormitum) preceding sleep in patients referred for insomnia or complaining of muscular jerks also during wakefulness. While in a regular 30-s PSG epoch the myoclonus-like activity in these muscle channels might appear simultaneous, in a more widespread temporal window (e.g., 5-s epoch) it became visible that the onset of the EMG activity was in the mid-thoracic or mid-abdominal muscles and subsequently spread rostrally and caudally. Neurophysiologically, Vetrugno and co-workers were able to demonstrate that in patients afflicted with involuntary jerks when falling asleep, the spread of this jerking activity was of the propriospinal type, with a spinal propagation velocity ranging from 2 to 16 m/s [29]. The authors also showed that it was necessary to perform PSG with multichannel surface EMG recording in these patients, which includes cranial nerve innervated muscles (which are usually not affected), intercostal and paraspinal muscles, as well as upper and lower extremity muscles. They showed that this activity was mostly present during pre-sleep wakefulness and in the transition to light sleep [29].

In addition, in daytime movement disorder clinics, it has been found that in many cases the propriospinal myoclonus represents a voluntary phenomenon, based on the fact that it is preceded by a Bereitschaftspotential [30]. It might be speculated that also in patients with suspected propriospinal myoclonus at sleep onset some cases might have a psychogenic origin. However, this can only be definitely ruled out with back averaging and clear documentation of the absence of a Bereitschaftspotential.

### Diagnostic criteria

In the ICSD-3, PSM at sleep onset is now listed among the category of SRMD and is thus recognized as having a definite pathology (as opposed to e.g. EFM and ALMA, which are listed as isolated symptoms). The ICSD-3 criteria for PMS are listed in Table 2. The criteria represent clearly a clinical definition and no minimum amount of jerks or no minimum requirement of EMG channels has been defined. Therefore, it seems plausible that, even following ICSD-3, functional cases may be misdiagnosed as true PSM [30, 31•, 32•].

**Table 2:** ICSD-3 criteria for propriospinal myoclonus [1]

ICSD 3 criteria for propriospinal myoclonus	
• The patient complains of sudden jerks, mainly of the abdomen, trunk, and neck	
• The jerks appear during relaxed wakefulness and drowsiness, as the patient attempts to fall asleep	
• The jerks disappear upon mental activation and with onset of a stable sleep stage	
• The jerks result in difficulty initiating sleep	
• The disorder is not better explained by another sleep disorder, medical or neurological disorder, mental disorder, medication use, or substance use disorder.	

### Differential diagnosis

PSM at sleep onset should be distinguished from intensified hypnic jerks and PLM. Hypnic jerks are very common and represent a physiological condition, but rarely they may be so frequent and severe to cause insomnia, a condition known as intensified hypnic jerks (IHJ) [33]. They present different types of motor pattern [34] and can originate in the cranial muscles spreading without any particular propagation pattern [35]. These features allow differential diagnosis between IHJ and PSM at sleep onset. PLM may be considered as a mimic of PSM, however, they appear with a periodic or pseudo-periodic pattern and involve mainly the distal limbs, without a propriospinal propagation pattern.

### Prevalence

True PSM at sleep onset is considered to be a rare condition. However, epidemiological data are lacking [1].

### Clinical relevance/meaning

In propriospinal myoclonus in general, which is often also intensified when laying down, it has been demonstrated that few cases are due to a structural lesion in the myelon, but the majority are considered functional movement disorders [30, 32]. In functional propriospinal myoclonus, with back averaging a Bereitschaftspotential preceding the jerk can be found and the jerks cease with sleep [30, 32].

### Treatment

No guidelines are available regarding treatment of true PSM at sleep onset. Single reports or case series reported amelioration of symptoms on treatment with clonazepam [36–38]. Valproate, zonisamide, levetiracetam, and opioids may improve symptoms in single cases [31•, 32•, 36].

Improvement of secondary propriospinal myoclonus has been described after the treatment of the underlying condition in few cases [39–43]. Patients with functional propriospinal myoclonus can benefit from unconventional therapy, such as autogenic training and biofeedback [44, 45].

## Isolated symptoms and normal variants within SRMD

In the ICSD-3 [1], this subcategory comprises hypnic jerks, EFM, HFT, and ALMA. While these were a separate category named “isolated symptoms, apparently normal variants, and unresolved issues” in the ICSD-2 [46], the ICSD-3 has included them in the SRMD category, as “isolated symptoms and normal variants” [1]. This might perhaps be subject to change or update in the next versions due to the evidence given below.

### Excessive fragmentary myoclonus of sleep

#### Overview

Fragmentary myoclonus in NREM sleep was first described by Broughton and Tolentino in 1984 in a single patient [47]. The next year, Broughton and co-workers published a series of 38 cases, which they determined to have EFM, defined ad hoc as “presence of EMG activity consisting of brief, usually less than 150 ms, hypersynchronous, potentials exceeding 50 μV in amplitude” recorded during at least 20 consecutive minutes of stage 2, 3, or 4 sleep, with a rate of at least 5/min [48]. Montagna and co-workers in 1988 described a similar phenomenon, which they defined as physiological hypnic myoclonus and observed that these findings were present also in wakefulness and often resembled fasciculation potentials [49].

#### Diagnostic criteria

According to the AASM scoring manual [50], the following define EFM: a. The usual maximum EMG burst duration seen in fragmentary myoclonus is 150 msec; b. At least 20 min of NREM sleep with EFM must be recorded; c. At least five EMG potentials per minute must be recorded. Here, a caveat needs to be mentioned that the current criteria of EFM are based exclusively on an ad hoc definition by Broughton and colleagues, who had reviewed 20-min segments of NREM sleep PSG in a series of 38 patients [48] and defined EFM as described above.

EFM is usually an incidental finding in the PSG and typically not associated with visible movements, although the ICSD-3 reports that there may be minor movements of the corners of the mouth, fingers, or toes [1]. In the AASM scoring manual [50], wording is slightly different: “in many cases no visible movements are present. Gross, jerk-like movements across the joint spaces are not observed. When minor movement across a joint space is present, the movement resembles the small twitch-like movements of the fingers, toes, and the corner of the mouth intermittently seen in REM sleep in normal individuals”.

#### Differential diagnosis

Differential diagnosis of EFM includes: i. phasic EMG activity in RBD. This is by definition present in REM sleep; however, the differential diagnosis is not always easy as EFM may be more intense during REM sleep as compared to NREM sleep; ii. propriospinal myoclonus, which is characterized by longer potentials as compared to EFM, and by a typical propagation pattern, as described before; iii. Head jerks/neck myoclonus (described below), which can be usually identified by the presence of short “stripe-shaped” movement-induced artifacts [51].

#### Prevalence

Our own group has published in 2011 a study on fragmentary myoclonus in sleep in 62 patients and found that the phenomenon was ubiquitous. It was not primary sleep related, but present both in sleep and wakefulness instead [52], in line with a previous finding by Montagna and co-workers [49]. In our cohort, most patients had low fragmentary myoclonus index and few patients had very high rates [52]. In 100 healthy sleepers, EFM was scored according to AASM criteria [50]. Every subject had FM during sleep. The median FM index during sleep was 25.5/h [19].

#### Clinical relevance/meaning

Based on the presence of EFM in both sleep and wakefulness, as previously shown by the Bologna group [49], we hypothesized that EFM during PSG was just an epiphenomenon of another different underlying pathology. We, therefore, performed EMG/NCV analysis in 98 out of 100 patients with EFM and found that 50% of them had electrophysiological abnormalities (namely polyneuropathy, nerve root lesions, and benign fasciculations) [53•]. Therefore, we suggested that EMG/NCV analysis should be performed in subjects with incidental finding of EFM during PSG to investigate the presence of peripheral nerve pathology [53•].

Furthermore, we would like to suggest that our finding that 9% of healthy normal sleepers meet the criteria for EFM [19] probably implicates that these criteria need to be reconsidered as they might turn out to be too liberal to distinguish physiological from truly excessive cases.

#### Treatment

The phenomenon represents an incidental EMG finding on PSG. Patients are usually not aware of fragmentary myoclonus and do not complain, therefore no treatment is needed. It has been described that in most cases, the cause appears to be benign [1]; however, the recent report of an association with electrophysiological abnormalities in 50% of subjects with EFM [53•] suggests that further studies are needed before classifying definitely this condition as a normal variant without clinical implications.

### Other minor motor activity during sleep

#### Overview and diagnostic criteria

HFT, RFM, ALMA, and HFLM represent minor motor activity during sleep with unclear significance and have somewhat overlapping features.

The term HFT has originally been created by Broughton in 1988 [33]. The authors described in two patients with severe head injury grouped phasic tremor potentials at varying frequencies between 0.5 and 1.5/s, recorded independently from both anterior tibialis muscles, occurring during pre-sleep wakefulness and sleep stages 1 and 2 [33]. In 2001, Wichniak and co-workers described this phenomenon as RFM while falling asleep [54]. RFM was found in 28/375 subjects (7.5%) and was described as rhythmic, oscillating movements of the whole foot or toes. In the EMG recordings, RFM was characterized by a series of repetitive phasic bursts of 300–700 msec with a frequency of 1–2 Hz, mostly occurring as single series with a duration of 10–15 s. RFM was present mainly during pre-sleep wakefulness and persisted in sleep stages 1 and 2 [54].

In 2003, Chervin and co-workers described a very similar phenomenon as ALMA. They reported a brief activation of the anterior tibialis in one leg alternated with similar activation contralaterally, with a movement duration of 0.1–0.5 s and a frequency of 1–2 Hz, with sequences lasting between several and 20 s. The phenomenon was particularly frequent during arousals [55].

HFLM have been defined by Yang and Winkelman [56] in 2010 as at least four consecutive discrete EMG bursts of leg muscle activity (unilateral, bilateral, or alternating) with a duration between 0.1 and 0.5 s and a frequency of 0.3–4 Hz. HFLM sequences were found in 33% of healthy sleepers [19]. In this cohort, the median number of HFLM sequences was 2 (range 1–24), with a mean HFLM sequence duration of 3.4 s (range 1.4–17.5 s) and a frequency of 0.9–4 Hz. There was no influence of age or sex on HFLM, but a moderate correlation with PLMS index > 5/h [19]. These data, taken together with the association between HFLM and RLS complaints reported by Yang and Winkelman [56], and with the EMG similarities between PLMS and HFLM, might suggest that HFLM are part of the broad PLMS spectrum [19].

#### Differential diagnosis

HFT, RFM, ALMA, and HFLM should be distinguished from other SRMD including PLM and PSM at sleep onset. Moreover, they should be distinguished from other movement disorders including painful legs and moving toe, tremor (e.g., in the context of Parkinson disease), and akathisia [1].

#### Prevalence

In a cohort of healthy sleepers, HFLM sequences were found in 33%. No influence of age or sex on HFLM was reported [19]. Epidemiological data on HFT, RFM, and ALMA are lacking.

#### Clinical relevance/meaning

It can be speculated that these overlapping minor motor activity during sleep may represent voluntary or unconscious stereotyped movements when falling asleep or during arousals, performed to relieve unpleasant sensations similar to the ones associated with RLS. If patients experience an urge to move, for instance due to a recognized or untreated RLS, it is considerable that the semiologic presentation might be similar. Therefore, RLS symptoms should always be specifically investigated in case of PSG findings of HFT, RFT, AMLA, or HFLM, as potential underlying condition causing these phenomena [57].

#### Treatment

As clinical relevance of HFT, RFT, ALMA, and HFLM is uncertain, and these SRMD are usually an incidental PSG finding and do not cause sleep disturbances, no treatment is indicated.

## Neck myoclonus/head jerks

### Overview and diagnostic criteria

Neck myoclonus during REM sleep has been defined and systematically investigated by the Innsbruck group in 2010. It is characterized by typical “stripe-shaped” movement-induced artifacts visible vertically over the EEG leads in the PSG, with a duration up to 2 s. Using surface neck muscle EMG, neck myoclonus can be detected as an increase in EMG activity over the background, ending in the case of return to background EMG activity for > 0.5 s [51].

### Differential diagnosis

As neck myoclonus is mainly present during REM sleep, it has to be distinguished from other EMG activity and movements during REM sleep, mainly RBD, and REM sleep without atonia (RWA). RBD behaviors typically involve the limbs [5], although some patients are reported who had predominantly head jerks as an expression of RBD behavior during the video-PSG recording.

Interestingly, recently a periodic presentation of neck myoclonus during sleep (periodic neck myoclonus during sleep, PNMS) has been reported in three subjects, fulfilling periodicity criteria used for PLMS. In these subjects, PNMS was mainly present during NREM sleep, whereas neck myoclonus not presenting in periodic series was present during REM sleep in the same subjects. The authors suggest the PNMS may be linked to PLMS and share common pathogenetic mechanism [58], thus representing a distinct entity from neck myoclonus without a characteristic of periodicity.

### Prevalence

In the original work [51], in a cohort of 205 mixed sleep disorder patient REM sleep was screened for the characteristic “stripe-shaped” movement-induced artifacts. If such an artifact was present, the video was inspected for the presence of neck myoclonus. Neck myoclonus was found in 112/205 patients representing 54.6%. The index was 1 ± 2.8/h of REM sleep. Younger patients had a higher index, and neck myoclonus during REM sleep was considered a physiological phenomenon [51]. In a cohort of 100 healthy sleepers, 35% presented neck myoclonus, with a median index of 2/h of REM sleep (range 0.7–41.2/h of REM sleep). No age differences in the distribution of neck myoclonus were found in this study. Neck myoclonus indices were higher in men than in women [19].

### Clinical relevance/meaning

Neck myoclonus is considered a physiological phenomenon, part of the spectrum of physiological twitching during REM sleep. However, it might be hypothesized that it represents a prodromal phase of RBD, as no study by now investigated its potential evolution to full-blown RBD over time. Nevertheless, the inverse age-relation speaks against this hypothesis. On the other side, the frequency of neck myoclonus is higher in patients with RBD or RWA than in patients without RBD or RWA [51]. Evolution of neck myoclonus over time needs to be investigated in further studies, to clarify whether neck myoclonus definitely represents a physiological phenomenon and if or when its excessive appearance should rather be considered prodromal RBD.

### Treatment

Generally, no treatment is needed. Notably, in the original work, none of the 13 patients on clonazepam had neck myoclonus [51]. However, if clonazepam could be a potential treatment, e.g., in case of neck myoclonus causing clinical complaints, is not known.

## Important differential diagnosis: prodromal RBD and isolated RBD

Although RBD is a parasomnia and not formally classified as a SRMD, it represents an important differential diagnosis for most of the aforementioned SRMD. RBD is characterized by vocalizations, jerks, and/or motor behaviors during REM sleep, often associated with REM-related dream content, and is accompanied by the absence of the physiological muscle atonia during REM sleep [1]. Idiopathic RBD (iRBD), in most recent terminology clinically isolated RBD (iRBD), has been thoroughly reviewed in a recent update paper [5••]. In this context, the new terminology is important. The term (clinically) isolated RBD seems to be more appropriate, in light of the increasing evidence that iRBD is not only a harbinger of neurodegeneration [59, 60] but also that multiple biomarkers of neurodegeneration are present even in those patients with long-standing clinically isolated (previously named idiopathic) RBD [61••].

Another important new concept is prodromal RBD, based on increasing evidence coming from EMG and video studies. Prodromal RBD refers to an intermediate phase in the evolution from normality to iRBD, in which neurophysiological and behavioral findings on video-PSG and EMG are present but do not yet meet established diagnostic criteria for RBD [5••]. The concept of prodromal RBD [5••, 62] allows for the first time not only to exactly define when iRBD ends and converts into overt alpha-synucleinopathy but also to start delineating the transition from prodromal RBD to begin of iRBD.

While treatment recommendations exist for clinically isolated RBD [5••], at present there is no recommendation if prodromal RBD should also be treated. While by definition prodromal RBD is often milder than clinically isolated RBD, no studies have been performed, if prodromal RBD is also at risk for injuries, which would implicate a need for symptomatic treatment. Furthermore, future disease-modifying therapeutic strategies may not only target isolated RBD as high-risk population to develop future PD but also prodromal RBD, because intervening at an earlier stage of progress may potentially improve chances to halt or reverse pathologic processes.

## Conclusions

For clinicians dealing with neurologic disorders, it is important to be aware of other minor SRMD beyond the well-known main motor disorders of sleep (e.g., RLS, bruxism, rhythmic movement disorders, etc.). Minor motor disorders of sleep comprise PLMD, propriospinal myoclonus at sleep onset, EFM, HFT, RFM, ALMA, HFLM, and neck myoclonus/head jerks. They should be correctly diagnosed, distinguished from differential diagnosis, and understood in their potential clinical relevance, in order also to start an appropriate treatment if needed.
